# Migration and descent, adaptations to altitude and tuberculosis in Nepalis and Tibetans

**DOI:** 10.1093/emph/eoac008

**Published:** 2022-03-08

**Authors:** Stephen Corbett, Jin-Gun Cho, Evan Ulbricht, Vitali Sintchenko

**Affiliations:** 1 Centre for Population Health, Western Sydney Local Health District, Sydney, New South Wales 2151, Australia; 2 Faculty of Medicine and Health, Westmead Clinical School, The University of Sydney, Sydney, New South Wales 2006, Australia; 3 Parramatta Chest Clinic, Parramatta, Sydney, New South Wales 2150, Australia; 4 Department of Respiratory and Sleep Medicine, Westmead Hospital, Wentworthville, New South Wales 2145, Australia; 5 Sydney Institute for Infectious Diseases and Sydney Medical School, The University of Sydney, Sydney, New South Wales 2006, Australia; 6 Centre for Infectious Diseases and Microbiology-Public Health, Institute of Clinical Pathology and Medical Research, Westmead Hospital and NSW Health Pathology, Sydney, New South Wales 2145, Australia

**Keywords:** tuberculosis, high-altitude adaptation, migration, hypoxia

## Abstract

**Background:**

High rates of tuberculosis (TB) in migrants from Tibet and Nepal have been documented for over 120 years and were previously ascribed to poor living conditions in the places of settlement. Adaptations to altitude involving genes in the Hypoxia-Inducible Factor pathway are present in 90–95% of Tibetans and in Nepalis these allele frequencies increase by 17% with each 1000 m increase in altitude.

**Methods:**

We calculated the incidence of TB by country of origin in immigrants from South and East Asia in New South Wales (NSW), Australia between 2004 and 2018, and compared disease severity, site of infection, evidence of local transmission and prevalence of latent TB, among these groups.

**Results:**

The incidence of active TB was consistently higher among 30 000 Nepalese and 1000 Tibetans than among all other immigrants to NSW. Nepal was the only country of origin where TB incidence in immigrants was not significantly lower than the reported TB incidence in the country of origin.

**Conclusions and implications:**

High rates of TB among Nepalese and Tibetan immigrants in Australia are unlikely to be attributable to pre-existing disease or local acquisition. Phenotypic effects of high-altitude adaptations may include a dampening of inflammatory responses to hypoxia, an effect unmasked by descent to a normoxic environment. A corollary of these findings may be that hypoxia-induced inflammation limits TB progression, reconfirming previous explanations for the apparent efficacy of high-altitude sanatoria. If vindicated by subsequent research, these provisional findings could open new avenues into preventive and host-directed interventions for tuberculosis.

**Lay Summary:**

The incidence of tuberculosis among Nepalese immigrants to Australia and other people of Tibetan heritage who migrate to lower altitudes is very high. In these screened populations, pre-existing active TB or locally acquired infection are unlikely explanations. We suggest that adaptations to altitude combined with descent to higher oxygen levels in air at sea level may be contributing factors.

## INTRODUCTION

The World Health Organization (WHO) ranks tuberculosis (TB) incidence in Nepal and Tibet as moderately high with the most recent estimate in Nepal being 238 cases per 100 000 population [1]. Since the cessation of the civil war in Nepal in 2006, over 30 000 Nepalese people have migrated to Sydney, New South Wales (NSW), Australia. Many of these immigrants are students, and most come from the Kathmandu Valley, which has an average elevation of 1500 m.

By 2014, clinicians in Sydney noticed a number of cases of active TB disease in these recently arrived students despite them being screened for active TB prior to entry into Australia for work or study. These observations were confirmed in an initial analysis of TB incidence in Nepalese immigrants to Western Sydney. They had a significantly higher crude incidence rate of TB (i.e. 156/100 000) than all other immigrant groups, and Nepal was the only country for which incidence rates in emigrants to Australia was comparable to the WHO reported incidence rate in their country of origin [2].

This study extended the previous report and examined in more depth the cause and implications of persistently high rates of TB in recent Nepali immigrants to all of NSW in three ways. First, we calculated the age distribution and incidence of latent and active TB among immigrants from high incidence countries into NSW between 2004 and 2018. We also gauged the extent of local transmission from analyses of whole genome sequences of *Mycobacterium tuberculosis* isolates from patients diagnosed with TB in NSW over this period. Second, we critically reviewed the evidence on the impact of altitude and migration from high to lowland areas on TB incidence, focusing on populations which are known to possess genetic adaptations to altitude. Third, we synthesized some of the earliest research linking altitude and TB occurrence to the discoveries of the Hypoxia-Inducible Factor (HIF) and genetic adaptations to altitude in Tibetans involving genes linked to this factor. We suggest that both may have a role in modulating inflammatory responses and TB incidence at altitude and at sea level.

## METHODS

The following investigations were carried out as part of a public health investigation into high rates of TB among Nepalese immigrants:

### Incidence

We calculated the age-standardized and age-specific incidence rates of TB in NSW between 2004 and 2018 in immigrants from the nine countries with the highest number of TB notifications, and from Tibet, in NSW. We extracted all cases of TB between 2004 and 2018 recorded on the NSW Notifiable Conditions Information Management System (NCIMS). Age-stratified population numbers for people born in these nine countries and in Tibet in NSW were obtained from census data from 2006, 2011 and 2016. Age-specific incidence rates of TB in NSW for the period 2004–2018 were calculated using Australian 2011 census year populations. Incidence rates were age-standardized using the NSW population and reported for the three quinquennia. Rates were also compared to incidence rates in 2016 from country of origin. We also calculated TB incidence by the number of years since arrival in Australia. Incident cases by year since arrival in Australia between 2004 and 2018 were extracted from the NCIMS. Denominators for these rates were obtained from census data, which asks immigrants in what year they arrived in Australia. Numbers reporting having been in Australia from 1 to 10 years were summed from these data. No adjustment was made for mortality or out-migration.

### Characteristics of TB cases from Nepal, India and the Philippines

We conducted an in-depth analysis of TB cases in immigrants from Nepal compared to immigrants from India and the Philippines, the two countries with the largest number of TB cases in immigrants in NSW.

#### Prevalence of latent TB

We estimated the prevalence of latent TB in students undertaking health-related courses, and in prospective employees within the NSW health system, all of whom are required to have either a Tuberculin Skin Test (TST) or an Interferon Gamma Release Assay to ascertain their current TB status. We retrieved the results of this screening from the TB clinic in Western Sydney, one of four within the Sydney Metropolitan Area, for the calendar year 2017. TST results were classified as being greater or < 10 mm, and > 15 mm.

#### TB incidence in those with a TB undertaking

All permanent visa applicants and applicants planning to stay in Australia for at least 6 months must undergo a general health assessment prior to migration. This is conducted in their country of origin, and includes screening for TB via a medical history, physical examination and a chest radiograph. If there are chest radiograph changes suggestive of TB, three sputum smears and culture examinations are required to exclude active TB. If diagnosed with active TB, migrants are not permitted entry into Australia until they have undergone treatment and been proven free of active disease on subsequent examinations. Applicants with a known history of TB or an abnormal chest radiograph, but negative results on sputum smear and culture, are at an increased risk of developing active disease and must sign an agreement to comply with the requirements of post-migration follow-up. This places them under obligation to report to a local chest clinic for medical follow-up within 28 days of arrival in Australia. Kaushik *et al*. [3] recently linked information on 32 550 migrants who had a TB undertaking in NSW with a subsequent diagnosis of active TB. Incidence rates among those with a TB undertaking by country of birth were calculated. We obtained results for Nepal from the authors.

#### Indices of disease severity and virulence

We used a number of proxy measures for disease severity and virulence—the site of disease, the proportion of Acid Fast Bacilli smear and TB culture positivity at diagnosis, and the time between migration and disease occurrence. This last index was stratified into arrival before and after 2008 to account for the relatively recent initiation of migration from Nepal.

#### Evidence of local transmission

Evidence of local transmission of TB was inferred using two sources of data—from epidemiological links between notified cases established by public health investigations and contact tracing, and since 2016, by prospective whole genome sequencing of all *M.tuberculosis* isolates recovered from culture-confirmed TB cases in NSW. Isolates were sequenced as described previously [4]. A genetic cluster was defined as at least two isolates sequenced in NSW that differed by no more than 12 single nucleotide polymorphisms. The proportion of cases in clusters was estimated for Nepalese, Indian and Philippines born cases between October 2016 and March 2019.

### Statistics

Crude incidence rates of TB were calculated among immigrants to NSW from the nine countries in South and East Asia with populations in 2018 exceeding 15 000 persons, and for the small population of Tibetan immigrants in NSW. Confidence limits for these rates were calculated using a direct standardization tool [5], which uses a normal approximation of the binomial method to calculate confidence intervals [6]. For estimates of prevalence, the proportions of cases with a TB undertaking, extra-pulmonary TB and smear positivity we calculated the exact 95% confidence intervals [7]. For the differences in age and years between emigration and diagnosis, we calculated t-tests for independent sample means [8].

## RESULTS

### Age distribution and incidence of active disease

**Figure 1.**
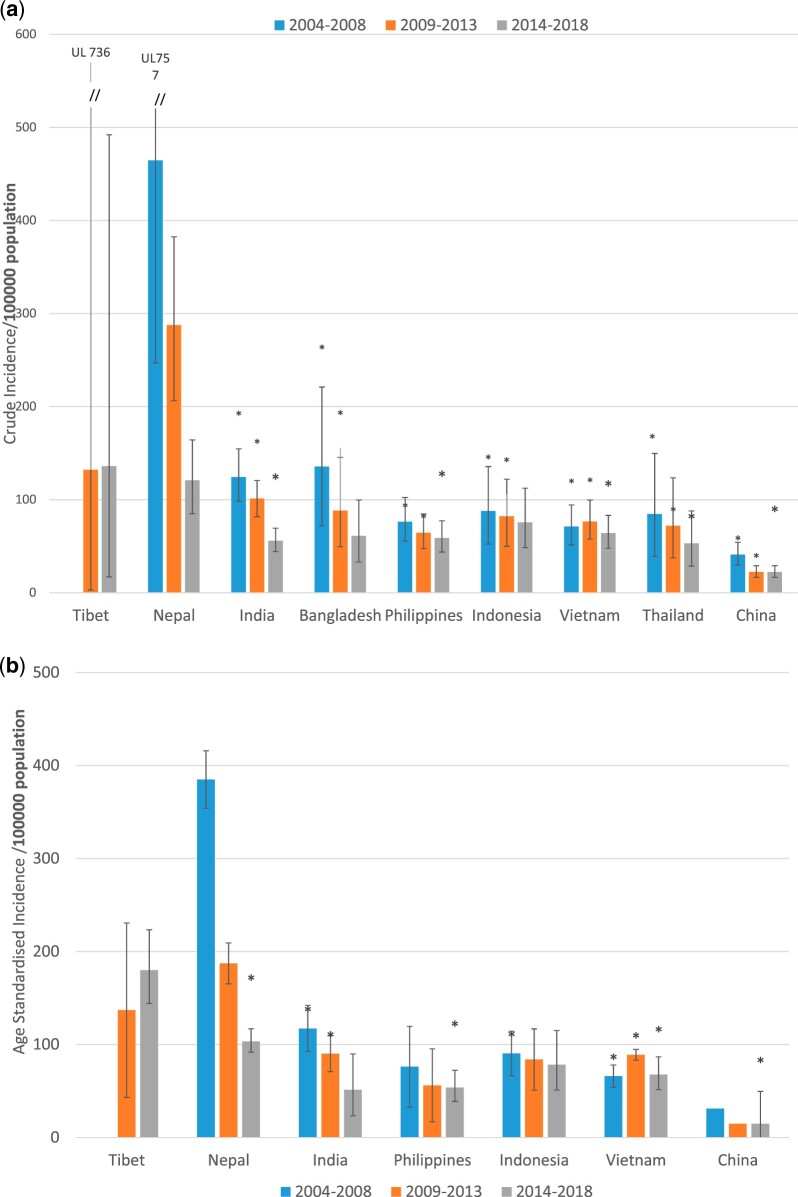
(**a**) Crude TB incidence rate/100 000 in immigrants to NSW from eight countries and Tibet, 2004–2018. (**b**) Age-standardized TB incidence rate/100 000 in immigrants to NSW from six countries and Tibet, 2004–2018

Crude TB incidence rates among Nepalese immigrants was significantly higher in each of three 5-year periods from 2004 to 2018 compared to immigrants from eight other countries in the region (Fig. 1A). Age-standardization reduced the significance of these differences (Fig. 1B), primarily because 85% Nepalese immigrants were in the 20–39 year age group which has a higher age-specific incidence. Age standardized incidence of TB in 2014–18 in the small Tibetan community in NSW of about 1000 people was even higher than in the Nepalese population. Small numbers of cases among Tibetans did not permit meaningful statistical comparison. Nepalese immigrants were significantly younger than immigrants from other countries in the region (Table 1).

**Table 1.** Characteristics of TB cases in immigrants from five countries, 2004–2018

	Nepal	India	Philippines	China	Vietnam
In-country TB incidence/10^5^ pop 2016	154	211	554	62	193
Cases in NSW immigrants	445	1142	626	651	726
Mean age (years)	35.9	45.5^***^	53.7^***^	62.2^***^	55.5^***^
Proportion female (%)	46	44	56	41	50
Diagnostics (%)					
Smear positive	34	26^*^	39	32	49^***^
Culture positive	66	60	77^**^	78^**^	86^***^
Site of infection (%)					
Lung	44	33^***^	54^**^	65^***^	58^***^
Lung + other site	11	9	9	8	8
Extra-pulmonary only	45	59^***^	37^*^	27^***^	34^***^
Migration to diagnosis (years)					
Migrated after 1 January 2008	2.6	2.3	2.7	2.4	2.7
Migrated before 1 January 2008	5.3	8.8^***^	16.3^***^	15^***^	18.6^***^
Conversion to active TB NSW 2000–2015					
Numbers on TB undertaking	420	3030	2775	7705	1535
% converting	4.5	2.5^*^	1.0 ^***^	0.7^***^	3.1

* Significant difference (*P* < 0.05) from Nepal.

** Significant difference (*P* < 0.01) from Nepal.

*** Significant difference (*P* < 0.0001) from Nepal.

**Figure 2.**
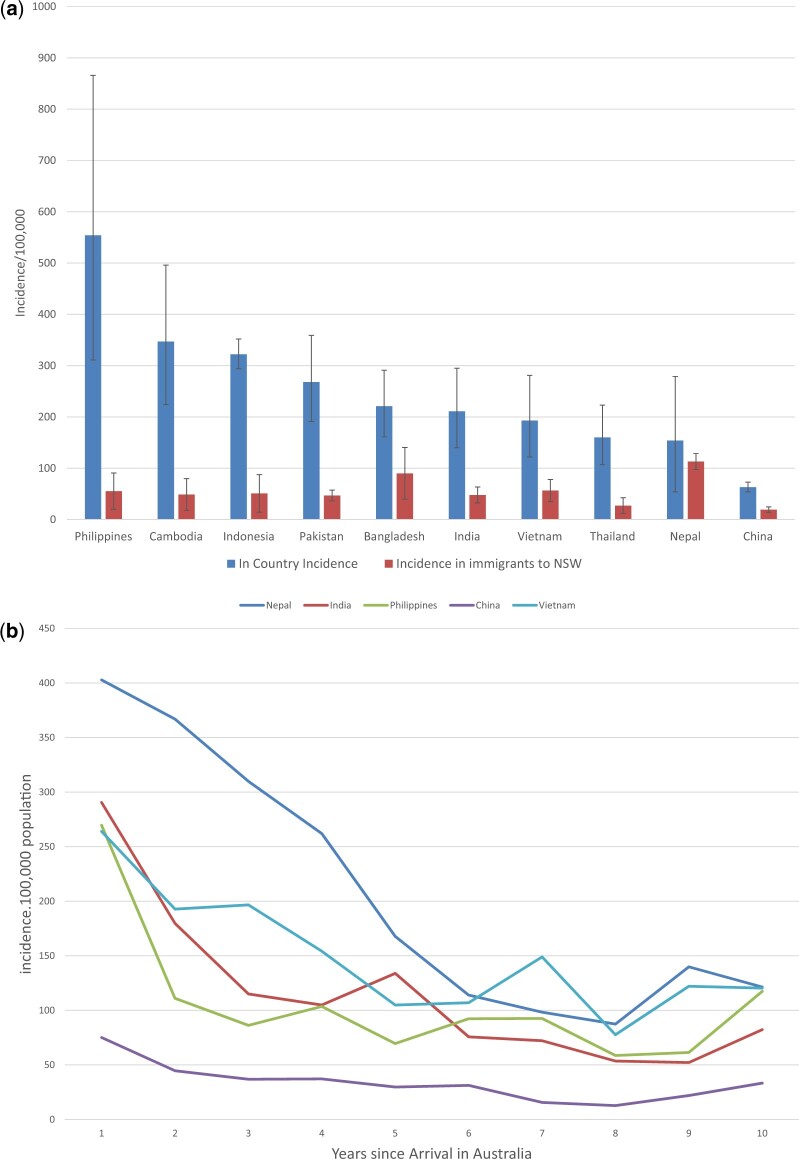
(**a**) Comparison of in-country TB incidence to incidence in NSW immigrants 2016. (**b**) TB incidence in immigrants to NSW from five countries, by number of years since arrival

In 2016, Nepal was the only country where the incidence in immigrants in NSW was not significantly lower than the TB incidence reported by WHO (Fig. 2A). The high incidence of TB among Nepalese immigrants was most evident in the 5 years following migration (Fig. 2B). Among the 32 550 migrants to NSW with a TB undertaking between 2000 and 2015, Nepalese immigrants had the highest rate of conversion (4.5%) to TB among all immigrant groups [3] (Table 1).

### Indices of disease severity

Forty-nine percent of Nepalese cases had extra-pulmonary TB, significantly more than immigrants from the Philippines, China and Vietnam and significantly less than immigrants from India. There were no differences in the proportion of disseminated or multi-organ TB among these immigrant groups. The differences between immigrants from Nepal, the Philippines and India in relation to the time to diagnosis were confined to those arriving prior to 2008. There were no differences in this measure in those arriving after 2008 (Table 1).

### Prevalence of latent TB

The prevalence of latent TB was highest among immigrants from the Philippines at 63%, compared to 47% and 49% among immigrants from India and Nepal, respectively. These differences were not statistically significant (Table 2).

**Table 2.** Prevalence of latent TB in students and health workers requiring mandatory TB screening, and genomic clustering of TB cases in immigrants from three countries

	Nepal	India	Philippines
Latent TB prevalence			
Number screened	174	194	100
TST > 10 MM %	49	47	63^*^
TST > 15 MM %	24	18	23
Whole genome sequencing			
No of TB cases	98	141	104
Clustering %	12	0.7^***^	6.7

* Significant difference (*P* < 0.05) from Nepal.

** Significant difference (*P* < 0.01) from Nepal.

*** Significant difference (*P* < 0.0001) from Nepal.

### Evidence of genomic clustering

Over 82% of all TB cases in NSW have been confirmed by *M. tuberculosis* culture where isolates have been available for whole genome sequencing. Analysis of genomes of *M. tuberculosis* recovered from culture-confirmed TB cases in NSW clustered in 12% of Nepalese cases, 7% of Philippine-born cases and, on average, 9% in all notified TB cases in NSW. All were significantly higher than the 1% of Indian-born cases that showed genomic clustering (Table 2). Of the 12 cases of TB among Nepalese immigrants which were linked genetically, 6 had confirmed epidemiological links which suggested recent local transmission after the migration to Australia.

## DISCUSSION

TB incidence among 30 000 mostly young people who have emigrated to NSW from Nepal since 2004 has been significantly higher than in immigrants from other countries in South and East Asia and not significantly different from the latest reported rates in Nepal itself. Nepalese immigrants have significantly higher rates in all age groups and higher rates of reactivation in those with a TB undertaking, and the effects are greatest in the 5 years following migration. Pre-migration screening and residence in a low-prevalence country significantly lowers TB incidence in all countries apart from Nepal. Even higher rates were observed in the small Tibetan community in NSW in the most recent quinquennium, although the number of observed cases were small.

The prevalence of latent TB in Nepalese immigrants is no higher than other immigrants and the small proportion of cases showing genetic clustering suggests that local transmission seems to be an unlikely cause of observed rates. These findings suggest that there are factors in either the host, the microbe or the environment, which make reactivation of latent TB more likely in Nepalese than in other immigrants in NSW.

**Figure 3.**
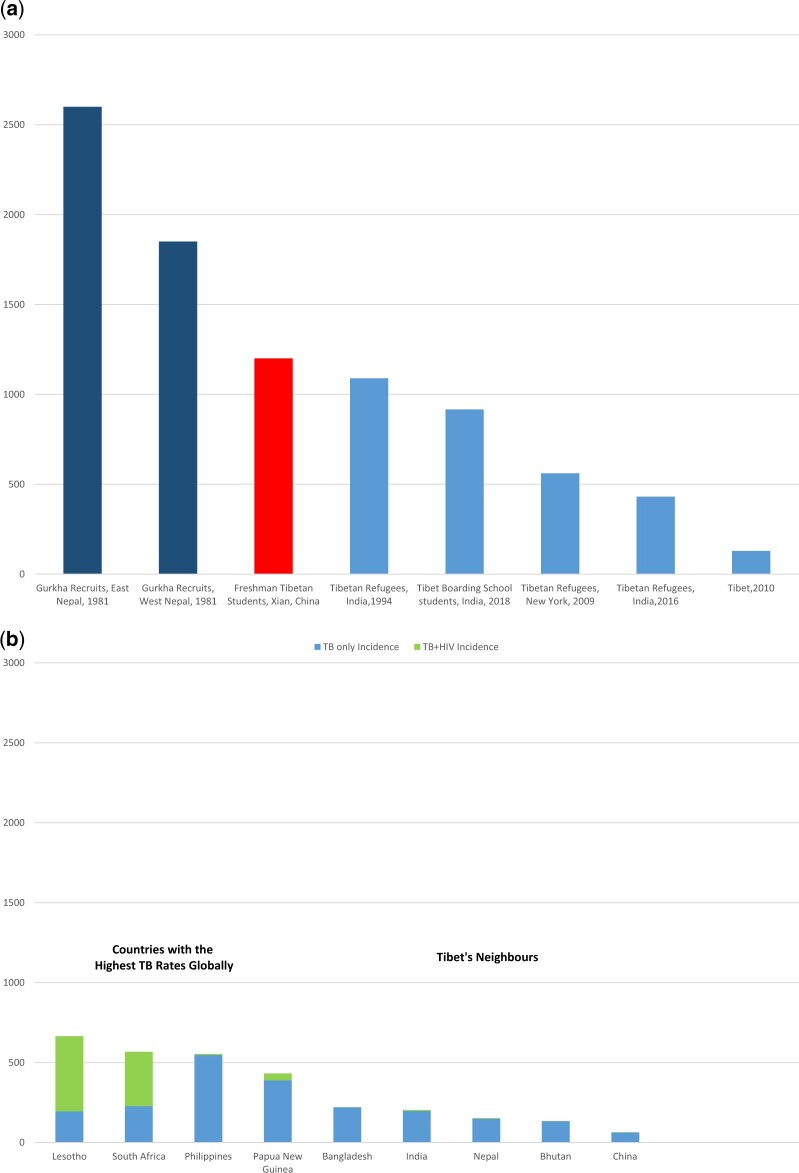
TB incidence in (**a**) Tibetans and Gurkhas who migrate to lower altitudes and (**b**) in high incidence countries and in countries neighbouring Tibet and Nepal [9–13]

Our findings reconfirmed previous reports about marked increases in the incidence of TB during migration of Himalayan populations of Tibetan heritage to lower altitudes. For over 125 years, high rates of TB have been observed in Nepalese recruits to Gurkha regiments in both the Indian and British armies [9]. Rates are highest among recruits from Eastern Nepal where the Rai and Limbu people have strong Tibetan heritage. In 1950, the People’s Liberation Army occupied Tibet and in 1958, the Chinese government established the Xizang Minzu University in Xian, Shaanxi Province (elevation 400 m) for students from Tibet and from other ethnic minorities. Rates of TB among Tibetan students are three times higher than in non-Tibetan students [10]. In 1959, the Dalai Lama fled Tibet and was followed by 100 000 Tibetans who settled in Dharamshala in India (elevation 1500 m). TB incidence among first- and second-generation Tibetans in Dharamshala remains high [11]. Tibetan refugees in Minnesota and New York [12, 13] have very high rates of latent (98% and 75% respectively) and active TB (Fig. 3).

Nepal as a country is a cultural mosaic comprising 103 caste and ethnic groups and with 106 language and dialect groups belonging mainly to the Tibeto-Burman and Indo-Aryan linguistic families. The Indo-Aryan language family constituted 79.1% of the population (48.6% Nepali, 12.3% Maithili, 7.5% Bhojpuri and 5.9% Tharu), and Tibeto-Burman 18.4% (5.2% Tamang, 3.6% Newari, 3.4% Magar, 2.2% Rai-Kiranti, 1.5% Gurung and 1.4% Limbu). This astonishing variety is indicative of the waves of migrations that have occurred for over 2000 years from the north and south of Nepal, respectively [14].

This ethnic and linguistic diversity is mirrored by recent genetic studies [15], which show that Nepalese share ancestry with their neighbours in South and East Asia. High altitude adaptations, which are likely to have originated only once in the Himalayan region, now show a marked cline in incidence in Nepal from low to high altitude.

Populations living at altitude in Ethiopia [16] and the Peruvian [17] and Bolivian [18] altiplano who migrate to low altitude in low TB prevalence countries also seem to be at a higher risk of sharp TB incidence in the years following migration, although to a lesser extent than is observed in Himalayan populations. In mountainous countries like Mexico [19], Peru [20], Vietnam [21], Nepal [22] and Papua New Guinea [23], highland provinces tend have lower rates of TB than lowland provinces and in Vietnam, these differences were shown not to be attributable to differences in detection [21]. Descent from high to lowland areas also increases TB incidence [24] but effects of descent itself are confounded by the effects of rural to urban migration, population density and poverty. All of these factors are likely to increase the risk of post-migration transmission of infection.

The impact of descent from altitude on the incidence of TB has received less attention historically than the beneficial effects of altitude on TB progression. Hermann Brehmer, who acquired TB as a medical student and was cured by living in the Himalayas, founded a high-altitude sanatorium for the treatment of pulmonary TB at Görbersdorf in Silesia in 1845. Similar institutions were established in Davos in Switzerland, where Thomas Mann set his great novel ‘The Magic Mountain’, in Denver in Colorado, Dokha in Nepal and other mountainous places. They became centres of TB treatment in an urbanizing century in which populations around the world were ravaged by this disease [25]. By 1953 in the USA, there were 839 institutions and over 136 000 beds [26]. This movement probably contributed to stemming the TB epidemic by removing infectious consumptives from crowded urban environments and the enforced rest and improved nutrition provided many compelling stories of recovery.

Past explanations for these altitudinal differences in TB incidence include the affinity of *M. tuberculosis* for oxygen, the impacts of ultra-violet light both on bacterial survival and transmission and Vitamin D synthesis at altitude, and hypobaric hypoxia. Kempner [27] first demonstrated a curvilinear relationship between oxygen uptake by mycobacteria and oxygen concentration and calculated that the inhibition of bacterial respiration would double at an altitude of 3000 m compared to sea level. By the 1930s, there was a consensus that *M. tuberculosis* was a facultative aerobic bacillus and that its oxygen consumption and growth were slowed considerably by exposure to hypoxia. *M. tuberculosis* cells respond to hypoxic conditions through activation of the DosR regulon, which in the hypoxic granulomas seen in latent TB, drives a non-replicative bacterial state that can be at least partially controlled by natural host immunity [28]. Descent from altitude may shift this delicate balance.

Ultra-violet radiation, which is bactericidal and increases in intensity 10–12% for each 1000 m of elevation, reduces the probability of TB transmission [29]. Vitamin D production is dependent on Ultraviolet B exposure, and Vitamin D deficiency is strongly linked to human susceptibility to TB [30]. Accordingly descent from altitude may reduce Vitamin D synthesis and render subjects more vulnerable to reactivation of their disease.

The French physiologist Denis Jourdanet (1815–92) ascribed the improvements in patients with pulmonary TB and other conditions at altitudes above 2000 m to reduced oxygen tension [31]. A report presented at the International Tuberculosis Congress in London in 1902 argued that the efficacy of treatment in a sanatorium increased with altitude. Dr Gerald Webb was the first president of the American Immunological Association and a member of the Anglo-American expedition to Pike’s Peak which described in detail the physiology of acclimatization to altitude. He moved to Denver, Colorado because his wife was diagnosed with TB and there established the American Climatological Association, which was very active in their investigation of the health effects of altitude. He established that there was an absolute and sustained increase in lymphocytes, but no overall increase in white cell count, in blood in humans and laboratory animals living at altitude [32]. He concluded that [33]:

‘… the increase in these cells (lymphocytes) brought about by altitude as being of such importance in the cure of tuberculous invalids and in the high degree of immunity our residents possess to this disease. We reason that the same factor which causes the increase in the red corpuscles in altitude must also originate the increase of the lymphocytes, namely marrow hyperplasia’

The discovery of HIF by Greg Semenza in 1995 has paved the way for a better understanding of these observations (William Kaelin, Greg Semenza and Peter Ratcliffe were awarded the Nobel Prize for Medicine in 2019 for this work). HIF transcription factors are a family of proteins that regulate the transcription of genes in response to falls in oxygen levels. *HIF-1, HIF-2* and *HIF-3* proteins all consist of an alpha sub-unit with corresponding genetic loci *HIF-1α, HIF-2α* (also known as *EPAS1*) and *HIF-3α.* These alpha subunits are produced continuously and degraded by interactions with another family of proteins called the prolyl hydroxylase domain proteins—*PHD1, PHD2* (also known as *EGLN1*) and *PHD3*. The latter are oxygen sensors which mark the *HIF-α* units for degradation under normoxic conditions. Under hypoxic conditions, the *HIF-α* units are stabilized and initiate transcription in hundreds of target genes [34].

HIFs are the master regulators of tissue and bodily responses to hypoxia including erythropoiesis, metabolism, angiogenesis and innate and adaptive immunity, and are directly involved in orchestrating changes involved in acclimatization to altitude. Acclimatization to altitude involves changes in the body in response to high-altitude hypoxia, the most important of which include increases in ventilation and in haemoglobin [35].

Hypoxia at altitude also invokes a HIF-mediated inflammatory response [36], which may influence the progression of TB [37, 38]. In most people, this inflammation is benign but in some contributes to the development of acute mountain sickness and high altitude pulmonary oedema. This inflammatory response includes lymphocyte proliferation, enhanced phagocytosis and neutrophil survival [39], more inflammatory and patrolling monocytes [40], the release of pro-inflammatory cytokines such as TNF-α and IFN-γ [36, 39], and angiogenesis [41].

Conversely hyperoxia, which can suppress inflammation [42], seems to be a risk factor for TB progression. Higher TB rates are observed in those living below sea level [19] or who have received treatment in a hyperbaric oxygen chamber [43]. South African gold miners work in some of the deepest mines and have some of the highest TB rates in the world [44]. They work in an extreme hyperoxic environment at 2 atmospheres of air pressure.

An ingenious experiment conducted in Peru in 2013 compared the effects of altitude on cellular immunity to effects on bacterial growth itself. The growth of modified luminescent Bacille Calmette-Guérin mycobacteria in whole blood was contrasted with growth in positive control culture broth and negative control serum at sea level and above 3400 m [45]. Ascent from low to high altitude was associated with increases in mycobacterial growth in culture broth and plasma but not in whole blood, suggesting that augmentation of anti-mycobacterial cellular immunity had a stronger effect than inhibition of mycobacterial growth [45].

These explanations for altitudinal differences in TB incidence do not, however, explain why people of Tibetan descent, who migrate to lower altitudes, should be at higher risk. We suggest that genetic adaptations to altitude by natural selection of genes in the HIF pathway are involved. The Tibetan plateau up to 4200 m had been settled by hunter-gatherers for 35 000 years, although permanent agricultural settlement dates back only to 5600–5900 years. Tibetans and Nepalis of Tibetan descent are adapted to living and working strenuously in an environment where the partial pressure of oxygen is between 50 and 60% of that at sea level. Physiological, anthropological and genomic research over the last 50 years has been able to characterize the phenotypic and genotypic differences between Han Chinese and Tibetans living at altitude. Phenotypically Tibetans have lower concentrations of haemoglobin at high altitude than the Han Chinese and have a higher hypoxic ventilatory response. Compared to Han Chinese, Tibetans living at altitude seem to be afforded protection from chronic mountain sickness and high altitude pulmonary oedema, strokes related to increased blood viscosity and low birth weight infants [46].

Genomic scans in Tibetans have identified over 40 genes in hypoxia and HIF pathways which show evidence of positive selection. Two genes are found consistently. The first is a haplotype of a Tibetan-selected *EPAS1* rs142764723 C/C allele that encodes the *HIF-2α*, and which remarkably, has introgressed into the human genome from an ancestral hominid, H denisova [34, 47, 48]. The second is a Tibetan-specific *EGLN1* haplotype encoding a *PHD2*^D4E; C127S^ variant. They are present in only 1–2% of Han Chinese lowlanders and are approaching fixation among Tibetan people. These two genes act in concert to blunten the erythropoietic response to hypoxia at altitude and at sea level [49] and may prevent the harmful effects of marked polycythaemia at altitude. Homozygotes for the *EGLN1* variant have a 12% reduction in haemoglobin level compared to Han Chinese subjects.

Recently, it has been shown that the inflammatory responses to hypoxia in Tibetans carrying *EGLN1* haplotype encoding a *PHD2*^D4E; C127S^ are also blunted compared to Han Chinese. In these Tibetans, there were less inflammatory and patrolling monocytes in circulation and reduced secretion of and induced chemotactic responses to cytokines IL6 and IL1β [40].

These findings may explain the high rates of TB that we and others have observed in people of Tibetan ancestry who descend to a normoxic environment. At high altitude, all humans are conferred some protection from the progression of tuberculous infection by the HIF-mediated inflammatory responses in the lung to hypoxia, as first proposed by Denis Jourdanet in 1863 [31] and Gerard Webb in 1909 [32]. This inflammatory response is diminished in Tibetans and Nepalis of Tibetan heritage who carry the *EPAS1* and *EGLN1* variants, evinced perhaps by the very high rates of latent TB seen in Tibetan emigrants to the USA and India.

**Figure 4.**
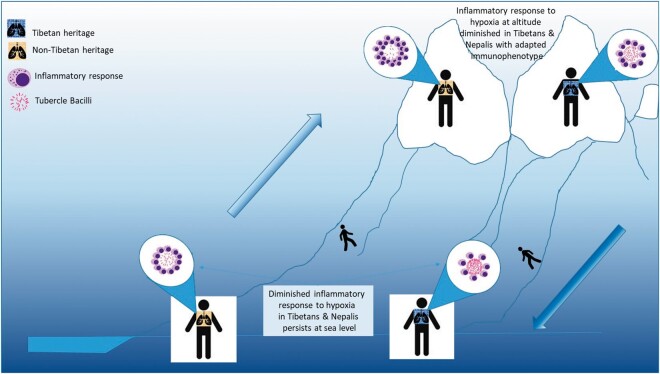
Schematic diagram comparing inflammatory responses to tuberculosis at sea level and at altitude among people of Tibetan and Nepali heritage and non-Tibetan and non-Nepali heritage. The HIF-mediated inflammatory responses to hypoxia is blunted in people of Tibetan and Nepali heritage

Descent to relative normoxia may, according to our hypothesis, achieve two things. First, it may reduce inflammatory responses provoked by hypoxia and foster the growth of the facultative anaerobe *M. tuberculosis*. Second, the presence of high altitude adaptations in some Nepalis may permanently impair their inflammatory responses to TB, as they seem to do with erythropoiesis—haemoglobin levels in Tibetans at sea level are 7% lower than in Han Chinese [49]. This explanation is illustrated schematically in Fig. 4.

## CONCLUSION

We have investigated possible causes of high TB incidence and prevalence among 30 000 Nepalis who migrated to Australia between 2004 and 2018. Despite being screened for TB prior to entry to a low TB incidence country, we have shown that they are at increased risk of reactivation of latent TB compared to immigrants from other countries. Our results are consistent with observations made over the last 110 years that Tibetans and Nepalis with strong Tibetan heritage are at an increased risk of TB upon migration to lower altitudes.

We suggest that two important factors contribute to this vulnerability to TB—adaptations to altitude involving the *EPAS1* and *EGLN1* genes in the HIF pathway and descent to a normoxic environment. These two factors may act in concert to reduce inflammatory responses to *M.tuberculosis*.

We have also conducted an assessment of the plausibility of our hypotheses using recently published data on the cline in prevalence of high-altitude adaptations in Nepal [15] (Box 1).

Plausibility of the hypothesis that high-altitude adaptations account for excess TB incidence in Nepalese emigrating to NSW The hypotheses advanced here rest on a number of assumptions: first that the EGLN1 variants are present among Nepali immigrants to Australia; second that these variants have a sufficient impact on the reactivation of TB to have produced the increases in TB incidence we have observed; and third that descent from altitude to a normoxic environment may also be associated with reduced inflammatory responses to TB. We have used some simple calculations to test the plausibility of these assumptions.

Between 2014 and 2018, there were 190 cases of tuberculosis diagnosed among 32 102 Nepalese people in NSW, a crude incidence rate of 120/100 000. If Nepalis had the same crude rate of TB as Indian people in NSW over the same period (55/100 000), they would have had 90 cases only, a difference of 100 cases which can putatively be attributed to the effects of high altitude adaptations or descent from altitude, or both. In-country incidence in India and Nepal are similar and our own survey suggests that the prevalence of latent TB in Indian and Nepalese immigrants is also similar—being about 50%.

Recent surveys [17] have shown that the allele frequencies of high-altitude adaptations in Nepal demonstrate a marked positive cline in incidence from zero to a maximum of 58%. Allele frequencies of these adaptations at 1500–2000 m of altitude are likely to range from 10 to 30%. Most immigrants and particularly students in Australia come from the Kathmandu valley with an elevation between 1500 and 2000 m.

We assume that phenotypic effects of these variants on cellular or innate immunity are confined to homozygotes for this variant, as is the case with haemoglobin [59,63]. We used the Hardy Weinberg formula to calculate the relative proportions of homozygotes and heterozygotes at each increment in altitude and allele frequency.

The relative risks of developing TB in largely homozygous Tibetans who migrate to Dharamshala or other provinces in China compared to the Indian or Han Chinese populations is between 5 and 10, although the magnitude of these risks may also be due to within group transmission.

Using this information, we calculated the mean number of cases in Nepalese which could be attributable to high-altitude adaptation for a range of allele frequencies under four different risk scenarios for homozygotes for the EGLN1 variants compared to a RR of 1 (no effect) for heterozygotes and those with no copies of the variant genes.

In Box 1, the cells shaded in yellow represent the range of allele frequencies for high altitude variants likely to be found in Nepalese immigrants to Australia. Cells shaded in orange show the levels of risk in homozygotes needed to account for the numbers of excess TB cases seen in Nepalese immigrants in NSW from 2014 to 2018.

**Box Table 1:**
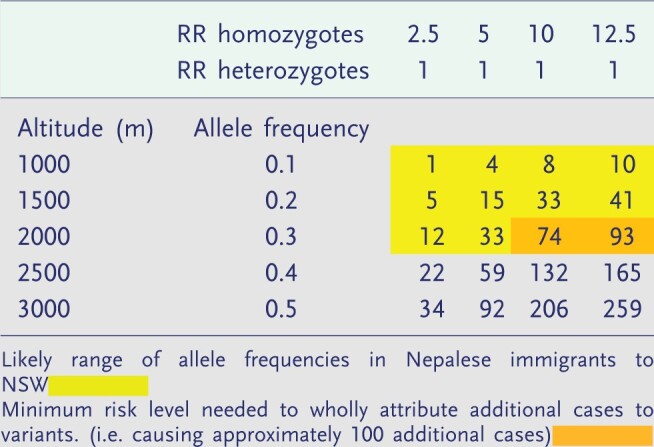
Estimations of additional TB cases in 32 122 Nepali immigrants in NSW population (compared to Indian immigrants) in the period 2014–2018, which could be attributable to high altitude adaptations in four risk scenarios over the range of allele frequencies

We conclude that the excess TB risks we have documented in Nepalese immigrants may partially (but probably not wholly) attribute to the excess cases of TB seen in Nepalese immigrants due to the presence of variant EGLN phenotypes.

The attenuation of inflammatory response to hypoxia conferred by high-altitude adaptations may also be beneficial in that it reduces maladaptive effects of acclimatization such as chronic mountain sickness. If acclimatization is maladaptive in the long term, then natural selection will tend to favour an attenuation of the induced phenotypic change [46].

This may also be an example of an evolutionary trade-off where a relatively small risk of mortality from TB is traded for a reduction in the effort of inflammation, which is metabolically expensive [50]. At present, the only indication we have that these mutations are implicated is that vulnerability to TB upon descent seems to increase with the degree of Tibetan heritage, as mentioned above.

There are two potentially important public health implications from our findings. There are 80 million people globally who live above 2500 m. At any one time, 3.5 million Nepalis and hundreds of thousands of Tibetans emigrate for work, study and economic opportunity to the Middle East, South-East Asia and Australia and to other provinces in China. For many, a diagnosis of TB is an economic catastrophe as it often involves repatriation and loss of income. Surveillance and prevention of reactivation of TB in these high-risk populations should be a public health priority. Second, hypobaric hypoxia has, as was postulated over a century ago, the potential to limit the progression of pulmonary TB.

The dual hypotheses presented here are consistent with our findings but remain provisional until confirmation by subsequent epidemiological and clinical studies. We have embarked on a case control study in Nepalese immigrants in NSW, which will examine the risks of carriage of these high-altitude mutations on TB risk while controlling for known socio-economic or dietary causes of TB reactivation such as nutrition or stress. Comparing TB risks in a Tibetans homozygous for high-altitude adaptations to heterozygotes would also be informative. We suggest that these studies could be part of a renewed focus on the beneficial effects of hypoxia and host-directed therapies which mimic the effects of hypoxia, and which have the potential to enhance TB treatment and control.

**Conflict of interest:** None declared. 
